# Supplementary material for: Predicting neuronal firing from calcium imaging using a control theoretic approach
Source: PLoS Comput Biol. 2025 Jun 19;21(6):e1012603. doi: 10.1371/journal.pcbi.1012603 (PMC12194039; doi:10.1371/journal.pcbi.1012603)
Supplement: S1 Appendix — (PDF) [file pcbi.1012603.s001.pdf]

## Supporting information

**S1 Appendix. Stability analysis.** In this appendix the governing ODE system (3) is shown to be stable for all realistic values of $s$. Begin the stability analysis by considering the intersection of nullclines. Let $\dot{x} = 0$, $\dot{z} = 0$. After a bit of algebra the coordinates of fixed point $(x^*, z^*)$ is found to be

$$(x^*, z^*) = \left( \frac{k_r z}{k_f (L - z)}, \frac{\alpha s k_f L}{\alpha s k_f + \gamma k_r} \right) = \left( \frac{\alpha s}{\gamma}, \frac{\alpha s k_f L}{\alpha s k_f + \gamma k_r} \right)$$

The Jacobian of the system is given by

$$J_f(x, z) = \begin{bmatrix} -\gamma - k_f \left( L - \frac{\alpha s k_f L}{\alpha s k_f + \gamma k_r} \right) & k_r + k_f x \\ k_f L - k_f \left( \frac{\alpha s k_f L}{\alpha s k_f + \gamma k_r} \right) & -k_f \left( \frac{\alpha s}{\gamma} \right) - k_r \end{bmatrix}$$

Examining the eigenvalues of this matrix upon evaluation at $(x^*, z^*)$, the condition put on s in order yield two negative eigenvalues, and hence stability, is simply that $s \in \mathbb{R}$. Note these bounds are found from numerical approximations, as an exact solution becomes intractable.

Since the range of $s$ is restricted to the positive reals, it follows that we have stability for all attainable values of $s$. To visualize that these ODEs produce a stable fixed point as a function of $s$ consider the following phase diagram, in which $s$ is fixed to be 30. Since our fixed point is a function of $s$, it follows that taking $s$ as a time varying function $s(t)$ would simply shift the location of our attractor. Taking parameters $\alpha = \gamma = 1, k_r = 7.6, k_f = 0.05135, L = 100, s = 30$, we would expect our fixed point to be at

$$(x^*, z^*) = \left( \frac{\alpha s}{\gamma}, \frac{\alpha s k_f L}{\alpha s k_f + \gamma k_r} \right) = (30, 16.854)$$

This is visualized in Fig A.
