# Supplementary material for: Predicting neuronal firing from calcium imaging using a control theoretic approach
Source: PLoS Comput Biol. 2025 Jun 19;21(6):e1012603. doi: 10.1371/journal.pcbi.1012603 (PMC12194039; doi:10.1371/journal.pcbi.1012603)
Supplement: S1 Appendix Fig A — (PDF) [file pcbi.1012603.s002.pdf]

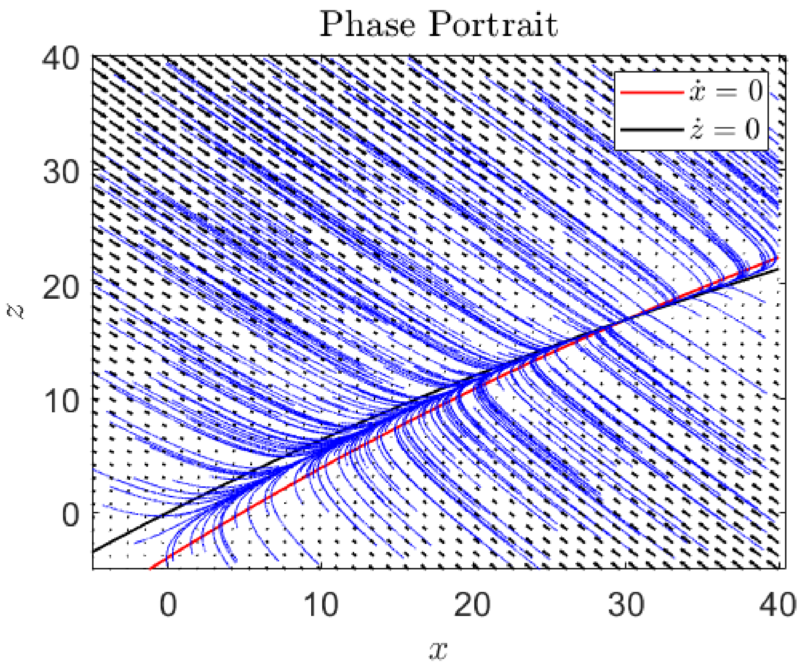

Fig A. Flows of equation (3). For this simulation, $\alpha = \gamma = 1$, $k_r = 7.6$, $k_f = 0.05135$, $L = 100$, $s = 30$. Note our fixed point is, as was expected, at $(30, 16.854)$.
