# Supplementary material for: Predicting neuronal firing from calcium imaging using a control theoretic approach
Source: PLoS Comput Biol. 2025 Jun 19;21(6):e1012603. doi: 10.1371/journal.pcbi.1012603 (PMC12194039; doi:10.1371/journal.pcbi.1012603)
Supplement: S2 Appendix — (PDF) [file pcbi.1012603.s003.pdf]

S2 Appendix. Auto-calibration of parameters, in particular α. In this appendix an auto-calibration process to infer reasonable parameter values, such as α, in equation (3) is detailed. This auto-calibration process requires a simultaneous recording of calcium and spiking activity as inputs. As few as 2 minutes of data suffices, provided a handful of spikes are present in the recording. Let z_{meas} denote the measured calcium concentration and s the measured spiking activity, both of which are functions of time. We may perform simple gradient descent on parameter vector $p = [\alpha, \gamma, k_r, k_f]$ as a function of the loss

$$l(p) = ||z_{meas} - z(p)||^2$$

Here $z(p)$ is computed by evolving the ODE system (3) to final time via numerical methods, including the dependence on p, but with the true s value supplied from the simultaneous recording. Then on gradient descent step k, we have for step size η

$$p_{k+1} = p_k + \eta \nabla_p \, l(p) \tag{5}$$

Notice for a recording up until final time T we have

$$\nabla_p \, l(p) = \int_0^T 2 \left(z_{meas} - z(p)\right) \left(\nabla_p \, z(p)\right) \, dt \tag{6}$$

and must now find a representation of $\nabla_p z(p)$. To do this, we leverage standard results from nonlinear dynamics which show the flow is of class C^∞ in both the parameters and time. Then Schwarz's Theorem allows us to take the gradient of both sides of equation (3)

$$\begin{cases} \frac{d}{dt} x &= \alpha s - \gamma x + k_r z - k_f x(L - z) \\ \frac{d}{dt} z &= k_f x(L - z) - k_r z \end{cases}$$

with respect to p, giving

$$\begin{cases} \frac{d}{dt} \nabla_p x &= \nabla_p \left(\alpha s - \gamma x + k_r z - k_f x(L - z)\right) \\ \frac{d}{dt} \nabla_p z &= \nabla_p \left(k_f x(L - z) - k_r z\right) \end{cases}$$

after swapping the orders of differentiation. This provides evolution equations for $\nabla_p z$ as desired, and we simply must solve the original ODE system coupled with this newly

created one to ascertain the missing term of (6). With this, we may perform gradient descent and find, at the least, a local minimum for our loss as function of p. This process may be carried out for all four parameters, but in experimenting with these systems allowing only control over α yielded the highest correlation scores. That is, we fixed k_f, k_r, γ and allowed only α to vary according to the evolution equations

$$\begin{cases} \frac{d}{dt}\nabla_\alpha x & = s - \gamma \nabla_\alpha x + k_r(\nabla_\alpha z) - k_f(\nabla_\alpha x)L + k_f\left(\nabla_\alpha x\right)z + k_f x(\nabla_\alpha z) \\ \frac{d}{dt}\nabla_\alpha z & = k_f(\nabla_\alpha x)L - k_f(\nabla_\alpha x)z + k_f x(\nabla_\alpha z) - k_r(\nabla_\alpha z) \end{cases} \tag{7}$$

Note this is a small abuse of notation, as in the above we have only one parameter we are considering, and as such are essentially just taking the derivative with respect to α. This can be performed for the other parameters, producing a new right hand side and set of equations for each.

One word of caution: The gradient descent process outlined above provides no guarantees on the positivity of α. In fact, if α goes negative, the system loses physical interpretability and will often become unstable. This is not a common occurrence, and was easily remedied by using the next neuron in the provided datasets. Should experimentalists encounter this in practice, we suggest moving to the a similar neuron and recalibrating. After 3 or so recordings a reasonable, positive α should be discovered.
