# Supplementary material for: Predicting neuronal firing from calcium imaging using a control theoretic approach
Source: PLoS Comput Biol. 2025 Jun 19;21(6):e1012603. doi: 10.1371/journal.pcbi.1012603 (PMC12194039; doi:10.1371/journal.pcbi.1012603)
Supplement: S1 Table — (PDF) [file pcbi.1012603.s004.pdf]

S1 Tables Parameter values and physical interpretation. The following Tables provide physical interpretation and units for various simulations based on governing equation (3).

Table A. Figure 1 parameters

Parameter	Value	Units	Description
α	16.66	μM/spike	Weighting of firing
γ	1.366	s^{-1}	Passive diffusion scalar
k_r	0.1	$\mu M^{-1}s^{-1}$	Forward reaction rate
k_f	10	s^{-1}	Reverse reaction rate
L	100	μM	Total indicator available
Δt	0.01	s	Simulation step size

Values provided below were utilized the in *spikefinder* simulations. α_i denotes the value of α that corresponds dataset i, found by the auto-calibration process in S2 Appendix.

Table B. Parameters and learned α values

Parameter	Value	Units	Description
α_1	22.80	μM/spike	Weighting of firing, dataset 1
α_2	10.31	μM/spike	Weighting of firing, dataset 2
α_3	45.71	μM/spike	Weighting of firing, dataset 3
α_4	7.90	μM/spike	Weighting of firing, dataset 4
α_5	14.75	μM/spike	Weighting of firing, dataset 5
α_6	32.60	μM/spike	Weighting of firing, dataset 6
α_7	53.33	μM/spike	Weighting of firing, dataset 7
α_8	55.83	μM/spike	Weighting of firing, dataset 8
α_9	21.71	μM/spike	Weighting of firing, dataset 9
α_{10}	10.76	μM/spike	Weighting of firing, dataset 10
k_r	0.2	$\mu M^{-1}s^{-1}$	Forward reaction rate
k_f	10	s^{-1}	Reverse reaction rate
γ	0.73	s^{-1}	Passive diffusion scalar
L	100	μM	Total indicator available
Δt	0.01	s	Simulation step size
