# Supplementary material for: Predicting neuronal firing from calcium imaging using a control theoretic approach
Source: PLoS Comput Biol. 2025 Jun 19;21(6):e1012603. doi: 10.1371/journal.pcbi.1012603 (PMC12194039; doi:10.1371/journal.pcbi.1012603)
Supplement: Attachment — Submitted filename: PLOS CB review.pdf [file pcbi.1012603.s005.pdf]

PLOS CB review

I like this paper, and the approach. I think more scientists knowing about MPC is a good thing, and this paper seems to be a nice application of those ideas (I've never actually used MPC before, though I have read about it). I have a few major comments. Below that I've pasted my stream of thoughts as I read the paper.

1. Other recently proposed methods for estimating spike times typically include a method to estimate parameters. This manuscript does not. The value of this work could therefore be substantially improved by simply adopting the unsupervised strategies proposed in other approaches (which are fairly agnostic to the spike inference procedures).
2. I want the figures to show more specific and direct comparisons. Eg, if there are 100 datasets, show 100 dots per algorithm, or show 100 dots per algorithm comparison (eg, MPC - Oasis).
3. Calcium to spikes is an inherently nonlinear and noisy process. Maybe this strategy results in better interpretability and insight, but maybe not. Any claims of improvement along some dimension, for me, require data-driven evidence to substantiate those claims. However, I see no such evidence, thereby, rendering such claims 'guesses', rather than empirically supported hypotheses.
4. Mentioning prior monte Carlo approaches for dealing with spike time inference from calcium imaging seems worthy.

Streaming comments:

- "State of the art methods depend largely on neural networks or non-mechanistic processes." Is non-negative deconvolution "non-mechanistic'? What is the threshold for considering a method 'mechanistic", and who gets to define that threshold? (Note: I'm biased in favor of NND, cuz I discovered its use for Ca imaging)
- The abstract mentions 'chemical reaction networks', but not MPC. It seems the main insight is to use MPC, so I'd think that goes in the abstract.
- Our paper on sequential Monte Carlo modeling of calcium imaging for spike detection seems highly relevant, but not mentioned, https://www.cell.com/biophysj/references/S0006-3495(09)00311-7. This is particularly weird since you do mention SMC as potential future work, so maybe you were unaware of that work. In any case, it is relevant. I

certainly would not require you to benchmark against it, cuz running the code was a bear, and who knows if it even runs anymore. But in terms of interpretability, it has been the best, to my knowledge. It also outperformed NND on many datasets, in my hands.

- In eq. 4, I'm confused by notation here. It seems like maybe CI without the time-subscript is CI at the end? But that is not how it is used in the paragraph above or the schematic above? I recommend denoting it with something like CI_\infinity, or something, to not overload and to clarify.
- I'm confused by Fig 1. Why is s_meas not binary? And what is the unit of time? And why are we talking about 'spike rate' in this manuscript, when you are finding spike times, not just rate?
- I'm also confused by Fig 2. How many datasets are there? Why are we summarizing, rather than reporting the result individually on each one, and comparing each method to MPC, which is a much stronger way to compare, and then one could compute a paired test to assess whether any are significantly better than another. Also, I'm surprised to see using both training and testing data in the same figure, I'd rather they be indicated as such, because that difference is quite important.
- Spikefinder is not the only available dataset with joint Ca and spikes. We used some in our papers, which came out before spike finder. I don't recall if all the data we used were included in spike finder or not, but I thought not, and those data are also available. So, it would be interesting to train on spike finder data for STM, and test on other data. Or, at least, if spike finder has multiple animals, maybe train on some, and test on others?
- Fig 3 & 4: I still want to see all the data, eg, with strip plots, rather than binned summaries like box plots and violin plots.
- Same deal for Fig 5: I'd rather see, for each dataset, a comparison between the two algorithms.
- For me, the issue of estimating the few parameters of the MPC approach is a non-neglible issue. An iterative automatic procedure, in which parameters are selected on a subset of the data, and evaluated on the remaining data (like Jewell did, for example, or our original NND approach), I think would be basically necessary to have people use this approach, in practice.
- I found the discussion on interpretability and "Biophysical Insights" overstated. I mean, maybe. But also, maybe not. There is no evidence from experiments that the insights and/or interpretations are correct. At this point, they are fine guesses. Maybe MPC can be used for this purpose in this setting, and also, maybe not. To me, this is more a discussion point for future work, rather than a 'result'.
