# Supplementary material for: Predicting neuronal firing from calcium imaging using a control theoretic approach
Source: PLoS Comput Biol. 2025 Jun 19;21(6):e1012603. doi: 10.1371/journal.pcbi.1012603 (PMC12194039; doi:10.1371/journal.pcbi.1012603)
Supplement: Attachment — Submitted filename: Response to Reviewers.pdf [file pcbi.1012603.s006.pdf]

Reviewer 1:

In this manuscript, the authors proposed an approach to infer neuronal spiking times from calcium imaging signals. By employing modern control architectures, specifically model predictive control (MPC), within a chemical reaction network framework, the authors demonstrated accurate, fast, and interpretable results for predicting firing times. Furthermore, this framework is not limited to inference but also offers insights into biophysical nuances through the hyperparameters of calcium indicators. In total, the study presents a useful modeling framework for inferring neuronal firing rates from calcium imaging traces. However, the results remain preliminary, and further in-depth studies are necessary to justify publication in PLOS Computational Biology.

Major Issues:

Accuracy issue. The authors compared their MPC algorithm to two state-of-the-art algorithms, STM and Oasis, in Figure 2. While the authors highlighted that MPC offers better interpretability than Oasis and requires less training data than STM, its accuracy is inferior. Given that accuracy is a primary concern for users converting calcium signals into spike trains, the performance of MPC should be further improved to enhance its practical utility.

To address this, we have implemented an auto-calibration functionality that improves our mean correlation score on the spikefinder challenge test data sets as well as alleviates the need for tuning by hand. Specifically, our mean score is now 0.4145 versus STM's 0.4244 - effectively a 0.01 difference in correlation. We hope this improvement in accuracy enhances the practical utility of our method to a satisfactory level in light of the other advantages pointed out by the reviewer. When using the Victor-Purpura distance our method outperforms Oasis on a subset of neurons.

We should also mention that due to an error published in a paper we referenced "Robustness of Spike Deconvolution for Neuronal Calcium Imaging" Reference number 9, the mean score for Oasis was originally incorrect and is now computed to be 0.58 when computed across all available datasets.

We emphasize in the manuscript now that our method provides the first known method that can be implemented for real-time feedback control without the need for variable backtracking. Oasis utilized a backtracking feature to adjust spiking event times and improve the quality of prediction. While correlation is high when the lookback is permitted to be more than 5 frames, permitting only 1 frame of lookback reduces correlation, as is seen in figure 6 of reference [7]. (https://journals.plos.org/ploscompbiol/article?id=10.1371/journal.pcbi.1005423)

Our approach enables event detection in real-time without updating past spike history based on incoming observations, which we hope justifies the method's practical utility further. We also offer suggestions on ways to improve the accuracy for future research.

We also wish to point out that in an application where feedback control is used to drive the cells towards a desired spiking pattern, the reference signal can be generated (replace CI**_meas with CI*_desired) and the MPC algorithm will drive neuronal stimulation to achieve the desired behavior. In this framework our method works out of the box with no lag.

A significant limitation of the MPC approach is its sensitivity to the chosen dataset. As seen in Figures 3 and 4, the correlation scores of MPC are inconsistent, with median values ranging from below 0.2 to nearly 0.6 depending on the dataset. Furthermore, the MPC method shows greater variability in its mean correlation scores compared to STM. This instability may lead to doubts about the reliability of results obtained using MPC.

Some variability witnessed is inherent in the spikefinder data provided. For example, the predicted spiking occurrences from dataset 9, neuron 2, produces a correlation of -0.0199 with respect to the MPC methodology. Oasis scores a -0.0193, and STM scores a -0.032. Means on this dataset are all significantly higher, leading us to believe this neuron's recorded ground truth data was skewed in some way. To better assess the variability in scores as it relates to the algorithm itself and not the quality of the dataset, we computed the standard deviation of correlation coefficient across the datasets for the different algorithms. Across all datasets, the standard deviation for STM is 0.127, for Oasis it's 0.178, and for our approach it's 0.173. The variability is comparable to Oasis, which is the state of the art.

We added the following table to the manuscript to summarize these findings:

	MPC	Oasis	STM
σ_1	0.064	0.069	0.065
σ_2	0.123	0.134	0.120
σ_3	0.118	0.143	0.112
σ_4	0.149	0.181	0.170
σ_5	0.109	0.129	0.125
σ_6	0.050	0.055	0.083
σ_7	0.092	0.079	0.079
σ_8	0.071	0.061	0.067
σ_9	0.163	0.176	0.138
σ_{10}	0.137	0.161	0.135
σ_{all}	0.173	0.178	0.127

The current implementation of the MPC model lacks robust auto-calibration functionality. Manual parameter adjustments are inefficient and require substantial biophysical knowledge from the user, making the model less user-friendly. Considering the noisy nature of calcium signaling data, parameter selection becomes even more challenging. Figure 7 highlights that the model's noise-resistance capabilities need further optimization to enhance usability.

As mentioned previously we have implemented an auto-calibration functionality that improves our mean correlation score on the spikefinder challenge test data sets as well as alleviates the need for tuning by hand.

We added a section to the appendix describing the approach in detail. We were excited to find that we only needed to train on 1-3 cells for each dataset for a relatively short period compared to the length of the experiment.

While the authors emphasized the availability of the MPC model for online control, Figure 6 showed temporal deviations between Ssim and Smeas, in particular for the first spike in the 10-second subset. If such deviations persist during online control, they could destabilize the closed-loop system. The authors should address whether these deviations impact real-time applications.

We thank the reviewer for this valuable comment. We acknowledge the reviewer's concern regarding temporal deviations between S_sim and S_meas for the first spike in the 10-second subset of fig 6, and note for more simple controller algorithms such as proportional control these temporal deviations would be problematic. However Model Predictive Control is uniquely designed to handle such discrepancies through its receding horizon approach, where the control policy is recomputed at each timestep based on updated measurements. This continual re-optimization ensures that transient deviations do not necessarily lead to instability in real-time applications. Previous studies [27] have demonstrated that MPC remains relatively robust against model imperfections and measurement noise, provided the system is appropriately tuned. In the context of this study, the observed deviations remain within expected tolerances and do not significantly impact closed-loop stability.

We added an additional reference and a subset of the above response to the manuscript to emphasize that this is not an issue with the MPC approach.

[27] Rawlings, J.B., Mayne, D.Q. and Diehl, M., 2017. Model predictive control: theory, computation, and design (Vol. 2). Madison, WI: Nob Hill Publishing.

The frequency response analysis pipeline described in the final section is undoubtedly valuable for understanding biological processes. However, it remains unclear how these biological insights translate into improved model performance. While the chemical reaction-based modeling

framework has strong biological interpretability, further optimizations are needed to enhance both the performance and practicality of the method.

Thank you for your insights. We agree with your argument, measuring how these biological insights translate into guidelines for engineering biomolecules is not properly validated. We believe to truly assess how these results translate into improved model performance, additional in vivo experiments would prove fruitful. However, we believe such an endeavor would be outside the scope of the paper since it's not the main contribution of our work. It would be a great direction for a future paper.

To address this comment, we have toned down the language to move away from unsubstantiated claims. This conjecture is now presented as such in the discussion section in hopes of generating further interest.

Minor concerns:

The full name of NND is missing from the text. Is it Non-Negative Deconvolution?

In line 253, the word "biophysical" is misspelled as "biophyiscal."

Thank you for catching these errors, we have fixed them.

Reviewer 2:

The manuscript introduces a novel method to predict neuronal firing rates from single cell calcium imaging data. The algorithm uses a mechanistic model based on chemical reaction networks (CRN) and model predictive control (MPC). This approach offers real-time computation capabilities and higher interpretability respect to the state of the art, especially as it is based on a model of calcium dynamics, addressing a key limitation of existing methods that rely heavily on neural networks or non-mechanistic strategies.

Major points:

As the topic of the study is the inference of spiking activity from calcium imaging traces, and since the vast majority of learning-based methods have been shown to be still outperformed by the much more simple nonnegative deconvolution (NND) 1, the authors correctly compare their method with NND.

The authors show that their method is outperformed by both the STM and NDD methods. While there is an argument that the efficiency of STM methods varies greatly depending on their training, this is not true for NND.

All in all, the method proposed by the authors offers improved interpretability at the cost of speed and accuracy, respect to NND. However, while I do find interpretability to be an extremely important factor in developing new methodologies, the target audience for this method is likely to care more about its predictive ability, and, secondarily, speed.

We thank you for this assessment of the landscape of methodologies. We are excited to share that we were able to increase the scores of the MPC approach by introducing an auto-calibration pipeline. This makes use easier, and increases scores, making it so the gap between STM and our methodology is less than 0.01 in correlation score. When using the Victor-Purpura distance our method outperforms Oasis on a subset of neurons.

However, we noticed an error in our analysis based on a misreporting of the Oasis scores, which we have now rectified. This gives Oasis a higher score than both STM and our approach. It is now computed to be 0.58 when computed across all available datasets.

We emphasize in the manuscript now that our method provides the first known method that can be implemented for real-time feedback control without the need for variable backtracking. Oasis utilized a backtracking feature to adjust spiking event times and improve the quality of prediction. While correlation is high when the lookback is permitted to be more than 5 frames, permitting only 1 frame of lookback reduces correlation, as is seen in figure 6 of reference [7]. (https://journals.plos.org/ploscompbiol/article?id=10.1371/journal.pcbi.1005423)

Our approach enables event detection in real-time without updating past spike history based on incoming observations, which we hope justifies the method's practical utility further. We also offer suggestions on ways to improve the accuracy for future research.

We also wish to point out that in an application where feedback control is used to drive the cells towards a desired spiking pattern, the reference signal can be generated (replace CI**_meas with CI*_desired) and the MPC algorithm will drive neuronal stimulation to achieve the desired behavior. In this framework our method works out of the box with no lag.

Minor Points:

I find it unclear why the authors refer specifically to two-photon calcium imaging, and not to calcium imaging in general. What is necessary to apply their analysis is to have calcium imaging at the single-cell resolution, not necessarily with a two-photon microscope. I see no reason why their analysis should not apply to 1-photon or multi-photon calcium imaging. Given the expertise of the authors with applied mathematics, I believe this might be since they have mostly cooperated with a laboratory that focuses on two-photon. I recommend the authors to adjust the manuscript accordingly, since their method is more widely applicable.

This is an excellent point - you are correct we were focused on 2-photon because of our relationship with a lab that employs such practices. We have updated the manuscript to reflect that the methodology may be applied to calcium imaging in general.

The requirement of having to tune the parameters for optimal performance, while being positive as it introduces an extra degree of control over the method, is similarly likely to constitute a potential source of error for least experienced experimenters.

We thank the reviewer for bringing this to our attention. To remedy this, we have introduced an auto-calibration functionality that infers reasonable parameters provided simultaneous calcium imaging and spiking activity, though we noted the highest increase in correlation score only varying α. In this way we retain the best of both worlds. That is, control over parameters allows experienced experimenters to play and gain valuable insights. On the other hand, the auto-calibration functionality alleviates this burden for the experimenter that wishes to simply plug and play without much consideration. We have added details of the method to the appendix.

Line 37, the authors explain for the second time the meaning of MPC, this is unnecessary as it has been explained already in line 28. I believe that instead, the STM acronym has not been explained.

This second explanation for MPC has been removed in Line 37. We have added the explanation for the STM acronym in the first instance where it appears.

Figure 2, what is NDD* in the legend?

Thank you for catching the typo. It should be NND*. NND* is the non-negative-deconvolution methodology Oasis, supplied with no information about the indicator. It represents the performance of NND when no information about the indicator is provided a priori. The caption in Fig. 2 is now accurate, and the legend is fixed.

Since the method seems very promising in taking into account different calcium indicator dynamics, it might be worth adding a comparison of performance with other methods in case of different calcium indicators (e.g. GCaMP6s vs GCaMP6f, or even better, RCaMP). It might potentially greatly improve the applicability of the method, since standard methods tend to struggle with RCaMP.

We find this suggestion interesting. However, the datasets are of varying quality and come from various types of experimental methods, including different animal models. Thus, with the current available dataset, it would be difficult to parse out performance based on the indicator.

https://pmc.ncbi.nlm.nih.gov/articles/PMC4888799/

Table 1.

Datasets

Set	Area	Brain state	n	Indicator	Scan frequency	Scanning method	#spikes	sp/s	Field of view
1	V1	AN	16	OGB-1	322.5 ± 53.2	3D AOD	19,876	1.86	200×200×100 μm^2
2	V1	AN	31	OGB-1	11.8 ± 0.9	2D galvo scan	32,385	2.47	250×250 μm^2
3	V1	AN	19* (11)	GCamp6s	59.1	2D resonant	23,974	2.58	265×265 μm^{2}135×135 μm^2
4	Retina	Ex vivo	9	OGB-1	7.8	2D galvo scan	12,488	4.36	100×100 μm^2
5	V1	AWK	15 (6)**	GCamp6s	59.1	2D resonant	12,413	4.87	265×265 μm^2

Reviewer 3:

I like this paper, and the approach. I think more scientists knowing about MPC is a good thing, and this paper seems to be a nice application of those ideas (I've never actually used MPC before, though I have read about it). I have a few major comments. Below that I've pasted my stream of thoughts as I read the paper.

Major comments:

Other recently proposed methods for estimating spike times typically include a method to estimate parameters. This manuscript does not. The value of this work could therefore be substantially improved by simply adopting the unsupervised strategies proposed in other approaches (which are fairly agnostic to the spike inference procedures).

Thank you for this specific suggestion which greatly improved our method all around and supported much of our needed revisions. We added an approach to auto-calibrate the model based on ground truth data from 1-3 neurons for a relatively short period of time. Details can be found in the appendix.

I want the figures to show more specific and direct comparisons. Eg, if there are 100 datasets, show 100 dots per algorithm, or show 100 dots per algorithm comparison (eg, MPC - Oasis).

Thank you for the suggestion to improve the presentation of the results. We have redone the plots as suggested.

Calcium to spikes is an inherently nonlinear and noisy process. Maybe this strategy results in better interpretability and insight, but maybe not. Any claims of improvement along some dimension, for me, require data-driven evidence to substantiate those claims. However, I see no such evidence, thereby, rendering such claims 'guesses', rather than empirically supported hypotheses.

Thank you for your insights. We agree with your argument, measuring how these biological insights translate into guidelines for engineering biomolecules is not properly validated. To address this comment, we have toned down the language to move away from unsubstantiated claims. This conjecture is now presented as such in the discussion section in hopes of generating further interest.

Mentioning prior Monte Carlo approaches for dealing with spike time inference from calcium imaging seems worthy.

Thank you for the suggestion, we added the following references to both the introduction and future work discussion section:

[3] Joshua T Vogelstein, Brendon O Watson, Adam M Packer, Rafael Yuste, Bruno Jedynak, and Liam Paninski. Spike inference from calcium imaging using sequential monte carlo methods. Biophysical Journal, 97(2):636–655, July 2009.

Below we respond to the general comments made by the reviewer if the comment was not already directly addressed in response to the specific minor and major comments above.

"State of the art methods depend largely on neural networks or nonmechanistic processes." Is non-negative deconvolution "nonmechanistic'? What is the threshold for considering a method 'mechanistic", and who gets to define that threshold? (Note: I'm biased in favor of NND, cuz I discovered its use for Ca imaging).

This is a thought provoking question. The authors consider a mechanistic model to be one where parameters and description of dynamics can be mapped to specific biological processes. There exists models with varying levels of abstraction such that the kinetics of multiple biological processes are lumped into one parameter. As we understand it, the NND method does in fact leverage a model. The utility of a model as a mechanistic model then depends on the biological insight that is sought vs the level of abstraction that is captured in the model. We have revised the framing of the contribution of this work. Primarily, we feel that it offers a unique approach to real-time detection of neuronal spikes without the need for backtracking, and with potential for improvement if combined with other approaches.

The abstract mentions 'chemical reaction networks', but not MPC. It seems the main insight is to use MPC, so I'd think that goes in the abstract.

This has been added to the abstract. Thank you!

Our paper on sequential Monte Carlo modeling of calcium imaging for spike detection seems highly relevant, but not mentioned, https://www.cell.com/biophysj/references/S0006-3495(09)00311-7. This is particularly weird since you do mention SMC as potential future work, so maybe you were unaware of that work. In any case, it is relevant. I certainly would not require you to benchmark against it, cuz running the code was a bear, and who knows if it even runs anymore. But in terms of interpretability, it has been the best, to my knowledge. It also outperformed NND on many datasets, in my hands.

Our sincere apologies for overlooking this work. Thank you for bringing this to our attention. We are especially interested to know that it has outperformed NND. We plan to look more into this approach and see if there is opportunity to leverage some aspect of it.

In eq. 4, I'm confused by notation here. It seems like maybe CI without the time-subscript is CI at the end? But that is not how it is used in the paragraph above or the schematic above? I recommend denoting it with something like CI_∞, or something, to not overload and to clarify.

This is a great catch - that notation did become overloaded. The terminal penalty function denotes the running difference between the measured and the simulated values. That is, if we've simulated until time T, that difference should contain $T/ \Delta t$ subtractions. I've included the subscript [0, T] and added some language about T to hopefully clarify and rightfully differentiate it from the above diagram. Thank you for this observation.

I'm confused by Fig 1. Why is s_{meas} not binary? And what is the unit of time? And why are we talking about 'spike rate' in this manuscript, when you are finding spike times, not just rate?

Thank you for pointing this out - s_{meas} is not binary as we plotted downsampled spikes, in accordance with the analysis of the authors of spikefinder. We have added language underscoring this to hopefully minimize similar confusion from other readers.

The unit of time is seconds, we've also added some language clarifying this. You are right anytime we have written spike rate, spike times would have sufficed. I believe since the post processed downsampled data is most commonly compared against, our vernacular leaned this way. The following text has been added to the body of the manuscript:

"Specifically, measured and simulated spiking signals are downsampled from their native rate of 10 ms by a factor of 4, yielding a bin width of 40 ms."

I'm also confused by Fig 2. How many datasets are there? Why are we summarizing, rather than reporting the result individually on each one, and comparing each method to MPC, which is a much stronger way to compare, and then one could compute a paired test to assess whether any are significantly better than another. Also, I'm surprised to see using both training and testing data in the same figure, I'd rather they be indicated as such, because that difference is quite important.

This is an excellent point - we were initially surprised by contemporary literature's willingness to do this as well. I believe since there are such a limited number of available datasets,

namely the spikefinder and CRCNS datasets that I am aware of, the train and test labels have been used in various ways throughout their life cycles to maximize benchmarking opportunities. Due to the impressively little amount of training data required (a single neuron from each of the 10 datasets for our methodology), we did include these in our figures. We would like to underscore the neurons on which we train do not score notably better than those we test on - the calibration process adjusts so few parameters we believe the method consistently underfits. Some exposition on the nature of train/test datasets is important nonetheless, we agree. This has been added to the manuscript.

Spikefinder is not the only available dataset with joint Ca and spikes. We used some in our papers, which came out before spike finder. I don’t recall if all the data we used were included in spike finder or not, but I thought not, and those data are also available. So, it would be interesting to train on spike finder data for STM, and test on other data. Or, at least, if spike finder has multiple animals, maybe train on some, and test on others?

We agree this would be an interesting avenue for future study - I have included a summary of the data available from the spikefinder challenge below. Spikefinder also provided the cai-1 dataset from crcns.org, which were labeled as datasets 6-10. I have done a bit of digging for additional simultaneous recordings on which to benchmark our methodology and am always hungry for more - should you be aware of where I can find the data in your aforementioned paper we would love to get our hands on it! The conditions of recording, such as animal and brain state, have not sufficiently been explored in our opinion, for any existing methodology.

Table 1.

Datasets

Set	Area	Brain state	n	Indicator	Scan frequency	Scanning method	#spikes	sp/s	Field of view
1	V1	AN	16	OGB-1	322.5 ± 53.2	3D AOD	19,876	1.86	200×200×100 μm^2
2	V1	AN	31	OGB-1	11.8 ± 0.9	2D galvo scan	32,385	2.47	250×250 μm^2
3	V1	AN	19[*] (11)	GCamp6s	59.1	2D resonant	23,974	2.58	265×265 $\mu m^2$135×135 μm^2
4	Retina	Ex vivo	9	OGB-1	7.8	2D galvo scan	12,488	4.36	100×100 μm^2
5	V1	AWK	15 (6)[**]	GCamp6s	59.1	2D resonant	12,413	4.87	265×265 μm^2

Fig 3 & 4: I still want to see all the data, eg, with strip plots, rather than binned summaries like box plots and violin plots. Same deal for Fig 5: I’d rather see, for each dataset, a comparison between the two algorithms.

This is an excellent point - per your suggestion we have added figures 3 and 7, which show a direct comparison between the algorithms under their respective metrics. These correspond to what is now Fig. 2 and 6. We thank you for elevating the quality of our presentation. With this new visual, we are able to see the set of neurons for which our method outperforms Oasis with respect to the Victor-Purpura distance. We did, however, leave the presentation of Fig. 4 and 5 the same, since the raw information is already in Fig. 3. We feel the current presentation offers a simplified summary of the breakdown across datasets.

For me, the issue of estimating the few parameters of the MPC approach is a non-neglible issue. An iterative automatic procedure, in which parameters are selected on a subset of the data, and evaluated on the remaining data (like Jewell did, for example, or our original NND approach), I think would be basically necessary to have people use this approach, in practice.

Thank you for prompting us to revisit this in our revision. We have arrived at an automated procedure for parameter tuning to carry out the algorithm as described in the response to the major comments.

I found the discussion on interpretability and "Biophysical Insights" overstated. I mean, maybe. But also, maybe not. There is no evidence from experiments that the insights and/or interpretations are correct. At this point, they are fine guesses. Maybe MPC can be used for this purpose in this setting, and also, maybe not. To me, this is more a discussion point for future work, rather than a 'result'.

Thank you, we agree with your comment and have reframed the discussion as such.
